# Activating transcription factor 3 (ATF3) regulates cell growth, apoptosis, invasion and collagen synthesis in keloid fibroblast through transforming growth factor beta (TGF-beta)/SMAD signaling pathway

**DOI:** 10.1080/21655979.2020.1860491

**Published:** 2020-12-23

**Authors:** Xue-Ming Wang, Xiu-Mei Liu, Yuting Wang, Zhen-Yu Chen

**Affiliations:** a Department of Plastic Surgery, Qingdao University, Qingdao, China; b Department of Plastic Surgery, Fujian Provincial Maternity and Children’s Hospital, Fuzhou, China; c Child Care Center, Fujian Provincial Maternity and Children’s Hospital, Fuzhou, China; d Department of Plastic Surgery, Yantai Yuhuangding Hospital, Yantai, China; e Medical Plastic and Cosmetic Center, The Affiliated Hospital of Qingdao University, Qingdao, China

**Keywords:** Keloid, proliferation, invasion, activating transcription factor 3

## Abstract

The successful treatment of keloids is a great challenge in the plastic surgery field. Activating transcription factor 3 (ATF3) is discovered as an adaptive responsive gene, which plays a critical role in fibroblast activation. This study aimed to investigate the expression and biological role of ATF3 in the pathogenesis of keloids. ATF3 expression in normal skins and keloids was evaluated by real-time PCR, western blot and immunohistochemistry. Effects of ATF3 on cell growth, apoptosis, invasion and collagen production were evaluated in keloid fibroblast cells overexpressing or downregulating ATF3. ATF3 expression was significantly elevated in keloid tissues when compared with that of normal skins and parakeloidal skin tissues. Moreover, ATF3 promoted cell proliferation and collagen production in keloid fibroblast cells. Conversely, transfection with siRNA targeting ATF3 led to decreased cell viability and collagen synthesis via inhibiting transforming growth factor-β1 (TGF-β1) and fibroblast growth factor 2/8 (FGF2/8) production in keloid fibroblasts. ATF3 could reduce the apoptosis rate of keloid fibroblast cells. Molecularly, we found that ATF3 promoted BCL2 level and inhibit the expression of BCL2 associated agonist of cell death (Bad), Caspase3 and Caspase9 in keloid fibroblast cells. ATF3 also enhanced the invasive potential via upregulating the expression of Matrix Metalloproteinases (MMP) family members (MMP1, MMP2, MMP9 and MMP13). ATF3 could induce activation of TGF-β/Smad signaling pathway in fibroblasts. Collectively, ATF3 could promote growth and invasion, and inhibit apoptosis via TGF-β/Smad pathway in keloid fibroblast cells, suggesting that ATF3 might be considered as a novel therapeutic target for the management of keloid.

## GRAPHICAL ABSTRACT

**Figure:**
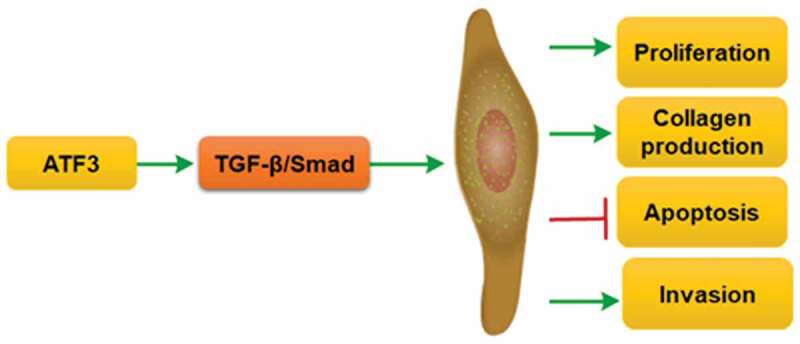

## Introduction

Keloid is one of the most common pathological scars produced from deregulated wound healing that occurs upon skin tissues damage [1]. Keloid is characterized by overproduction of extracellular matrix, excessive fibroblast proliferation, overexpression of growth factors and anomalous disposition of collagen fibers [2]. The main therapeutic modality of keloid includes surgery, drugs, laser therapy and radiotherapy [3]. However, the complete pathophysiology of keloid remains unclear.

Activating transcription factor 3 (ATF3) belongs to the ATF/CREB family. As a responsive gene, ATF3 is frequently elevated upon stimulation from a wide range of intra- and extracellular stresses [4,5]. ATF3 plays critical roles in regulating cellular behaviors by homo- or heterodimerizing with ATF members to activate or repress downstream genes [6]. Many studies have shown that ATF3 plays a vital role in cardiac fibrosis and apoptosis by modulating downstream signaling pathways, such as ERK1/2, JNK, and NF-κB [4,7]. Moreover, ATF3 promotes cell cycle transition by activating cyclin D1 expression in hepatocytes [8]. Changes in ATF3 expression are also associated with innate immunity response [9,10]. Lipopolysaccharide induced activation of TLR4 promotes ATF3, modulating the expression of various inflammatory genes, such as interleukin-6 (IL-6) and tumor necrosis factor-α (TNF-α) [11]. Particularly, accumulating evidence showed that ATF3 plays a critical role in fibroblast activation [12–14]. However, it remains unknown whether ATF3 could regulate the cellular behaviors of keloid fibroblasts.

Transforming growth factor-beta (TGF-β) could be abundantly released by injured tissues, and thus plays a critical role in pathological fibrosis, including keloid formation [15]. Firstly, TGF-β binds to type II TGF-β receptor (TβRII) which associates with and phosphorylates type I receptor (TβRI). Secondly, TβRI activates Smad signaling pathway to initiate a variety of physiological effects. Accumulating evidence revealed that TGF-β was dramatically elevated in keloid fibroblasts. A recent study has demonstrated a critical role of TGF-β/Smad signaling in the activation of keloid fibroblasts [16].

In the present study, we hypothesized that ATF3 is involved in the pathogenesis of keloids by activating TGF-β/Smad signaling. To prove such a hypothesis, we detected the expression profile of ATF3 in human keloid tissues and evaluated its effects on keloid fibroblast cell growth, apoptosis, invasion and collagen production. Additionally, the functional relevance of ATF3 and TGF-β/Smad pathway in activated keloid fibroblasts was investigated.

## Materials and methods

### Samples

Thirty paired keloids and their adjacent skin tissues were collected at the Medical Plastic and Cosmetic Center, the Affiliated Hospital of Qingdao University between December 2018 and December 2019. Healthy skin tissues (n = 30) were collected at the Medical Plastic and Cosmetic Center, the Affiliated Hospital of Qingdao University during the same period. The keloid patients were not treated with any drugs, hormones, and radiation before surgery. This study was approved by the ethics committee of the Affiliated Hospital of Qingdao University and all participants signed informed consent.

### Cell culture

The tissue was surgically removed, rinsed in PBS, and incised into small pieces of 1 mm size. They were digested by type I collagenase at 37°C for 4–6 h and subject to centrifugation at 800 x g for 10 min. Then, fibroblasts were collected and seeded to cell culture dishes in an incubator (5% CO_2_, 37°C). When the confluence reached above 90%, cells were trypsinized and passaged. After three or four passages, fibroblasts were used for experiments.

### MTT assay

Keloid fibroblast cells were seeded into 96-well plates at a density of 3.0 × 10^3^ cells per well in 200 mL DMEM medium for 12 h. Then, cells were transfected with negative control siRNA (siRNA NC), negative control plasmids (overexpression NC) and target gene-manipulated groups (recombinant plasmid encoding ATF3 and ATF3 siRNA). After 48 h of transfection, cell viability was detected by adding fresh medium containing 20 mL of MTT solution (5 mg/mL). After incubation at 37°C for 2 h, the optical absorbance was measured at 490 nm.

### Transfections

Transfections of plasmid DNA were performed using the Lipofectamine 2000 (ThermoFisher Scientific, Inc., Waltham, MA, USA) according to the manufacturer’s instructions. The ATF3 overexpression was conducted by constructing the full-length ATF3 sequences into cells via pLV-IRES-DsRed plasmids (GenePharma, Shanghai, China). The siRNA specific for ATF3 was purchased from RiboBio (Guangzhou, Guangdong, China). For gene transduction, fibroblasts were incubated with the viral media supplemented with 8 µg/ml polybrene overnight at a 37°C incubator supplemented with 5% CO_2_. Forty-eight hours after transfection, cells were harvested for subsequent experiments.

### Real-time PCR

Total RNAs were extracted and reversely transcribed into cDNA with a cDNA synthesis kit (Stratagene, USA), followed by the manufacturer’s recommendations. The PCR reaction was performed on the ABI 7300 Sequence Detection System (Applied Biosystems, Carlsbad, CA, USA). The primer sequences used were as follows: ATF3: 5ʹ- CAGAGCCTGGTGTTGTGCTA-3ʹ (forward) and 5ʹ-AGGTGTCGTCCATCCTCTGTT-3ʹ (reverse); TGF-β1: 5ʹ-AGAGCCATCGCCATAGTGTC-3ʹ (forward) and 5ʹ- AGAGACGCTGCCATTGATCC −3ʹ (reverse); FGF8: 5ʹ- AAGAGCTGGGCTAAGCCGTC −3ʹ (forward) and 5ʹ-GTGCCCGTCTGAAAACACCT-3ʹ (reverse); fibroblast growth factor 2 (FGF2): 5ʹ- ATCCACCAGAGCAAGAGTCC-3ʹ (forward) and 5ʹ- GGTCACAGGACTGA ACCACAT −3ʹ (reverse); collagen I (COLI): 5ʹ-CCTGAGAGGAGCATCAAGAGC-3ʹ (forward) and 5ʹ- AAGCGAGCAGAGAGGCTTAC −3ʹ (reverse); collagen III (COLIII): 5ʹ- TCCAGTTCCTTTCTG GCGAA −3ʹ (forward) and 5ʹ-AGGGCCTACCAAGAAGATGC −3ʹ (reverse); BCL2: 5ʹ- AGCTCCAGGGCTATCACTCA −3ʹ (forward) and 5ʹ- CATGG GCAGCTACTCGTCTT −3ʹ (reverse); Bad: 5ʹ-TAGTACTGGGTAGCCCTCGC-3ʹ (forward) and 5ʹ- CCGCTTGTTG GGGTCATAGT-3ʹ (reverse); Caspase3: 5ʹ- TGATAGCGTGCC ATGCAAAG-3ʹ (forward) and 5ʹ- GGGCTTGCGACAATCACAAC −3ʹ (reverse); Caspase9: 5ʹ-TCAGTGTGTGGGAG GACAGA-3ʹ (forward) and 5ʹ- AAGCTGAGCTACTCA GAG GGA-3ʹ (reverse); β-actin: 5ʹ- CTGTATGCCTCTGGTCGTAC-3ʹ (forward) and 5ʹ- TGATGTCACGCACGATTTCC-3ʹ (reverse). The amplification program included an initial denaturation at 94°C for 5 seconds, followed by 35 cycles of 30 seconds at 94°C, 30 seconds at 55°C, and 40 seconds at 72°C, and a final extension at 72°C for 10 minutes. The relative mRNA expression of each gene was calculated using 2(-ΔΔCt) with β-actin as the internal reference.

### Western blot

Total proteins from tissues or fibroblasts were lysed with lysis buffer. Equal amounts of protein samples (20 μg) were subject to a 10% SDS-PAGE gel and then transferred on to a PVDF membrane. Thereafter, the membrane was blocked with 5% nonfat dry milk in PBS for 2 h at room temperature. The membrane was incubated with the primary antibody against ATF3 (dilution, 1:1000, Santa Cruz Biotechnology, Santa Cruz, CA, USA), TGF-β1 (dilution, 1:1000, Santa Cruz Biotechnology, Santa Cruz, CA, USA), FGF8 (dilution, 1:1000, Santa Cruz Biotechnology, Santa Cruz, CA, USA), FGF2 (dilution, 1:1000, Santa Cruz Biotechnology, Santa Cruz, CA, USA), COI (dilution, 1:1000, Santa Cruz Biotechnology, Santa Cruz, CA, USA), COIII (dilution, 1:1000, Santa Cruz Biotechnology, Santa Cruz, CA, USA), BCL2 (dilution, 1:1000, Santa Cruz Biotechnology, Santa Cruz, CA, USA), Bad (dilution, 1:1000, Santa Cruz Biotechnology, Santa Cruz, CA, USA), Caspase3 (dilution, 1:1000, Santa Cruz Biotechnology, Santa Cruz, CA, USA), Caspase9 (dilution, 1:1000, Santa Cruz Biotechnology, Santa Cruz, CA, USA), and β-actin (dilution, 1:5000, Santa Cruz Biotechnology, Santa Cruz, CA, USA) overnight at 4°C. After washing for three times in TBST, the membrane was incubated with horseradish peroxidase-conjugated secondary antibody at room temperature for 2 h. The protein signals were detected using an enhanced chemiluminescence kit (ECL, Amersham) and quantified using Quantity-One software (Bio-Rad Laboratories, USA).

### Immunohistochemistry

The 4-mm paraffin sections were deparaffinized, rehydrated and heated in a microwave oven. The section was incubated with the primary antibody against ATF3 (dilution, 1:1000, Santa Cruz Biotechnology, Santa Cruz, CA, USA) at 37°C for 60 min, and then with secondary antibodies at 37°C for 30 min. After DAB coloration, images of the cell were observed under an Olympus microscope (Tokyo, Japan).

### Apoptosis assay

Keloid fibroblast cells (1.0 × 10^6^ cells) were harvested, washed, and fixed in ice-cold ethanol. Then, cells were re-suspended in the staining solution containing 5 µL of FITC-conjugated annexin V antibody and 5 µL of propidium iodide (BD Bioscience, San Jose, CA, USA). After incubation in the dark, the apoptotic cells were detected on a flow cytometer (BD Bioscience, San Jose, CA, USA).

### Transwell invasion assay

Keloid fibroblast cells (3.0 x 10^3^ cells) were cultured on the top of an 8-μm pore transwell chamber (Millipore, Billerica, MA, USA) pre-coated with Matrigel (BD Biosciences, San Jose, CA, USA). The lower chamber contained 500 μl of DMEM with 10% FBS. After incubating at 37°C for 30 min, cells in the lower surface of the membrane were fixed in methanol, stained with 0.5% crystal violet. The invasive cells were counted under an inverted microscope (Tokyo, Japan).

### Statistical analysis

Data were expressed as mean ± SD and subject to the SPSS 17.0 Windows version of software (SPSS). Differences between multiple groups were analyzed by one-way ANOVA followed by Dunnett’s multiple comparisons test. P < 0.05 was considered to be statistically significant.

## Results

### ATF3 expression is up-regulated in human keloid tissues

**Figure 1.**
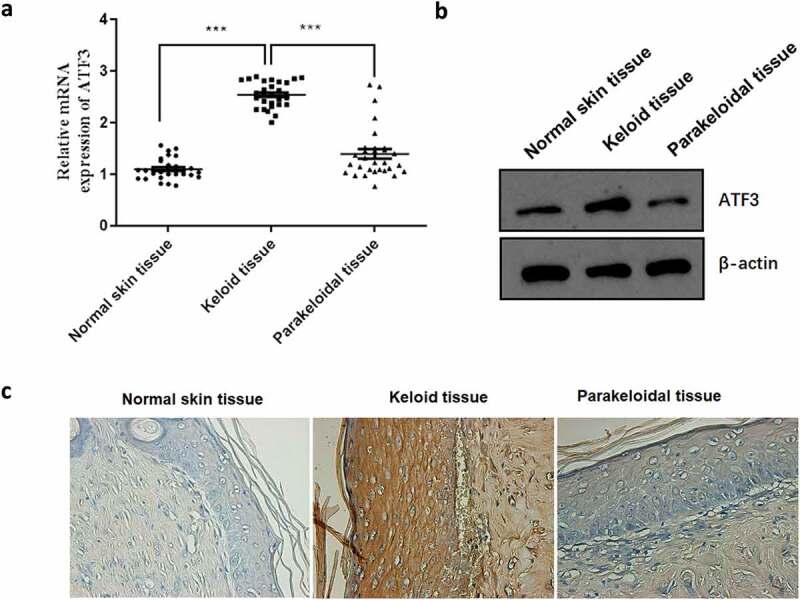
ATF3 expression is up-regulated in human keloid tissues

ATF3 has been reported to be involved in fibroblast activation [12,13]. In order to test the functional relevance of ATF, we firstly determined its expression profile in normal skins (n = 30), keloids (n = 30) and the corresponding parakeloidal tissues (n = 30). Data showed that the mRNA and protein expression of ATF3 was significantly elevated in keloid tissues when compared with that of normal skins and parakeloidal skin tissues (Figure 1(a,b)). Additionally, immunohistochemical analysis showed that the protein expression level of ATF3 was obviously up-regulated in keloid tissues (Figure 1(c)). These results validated the up-regulation of ATF3 in keloid tissues, suggesting that ATF3 may play a role in the pathogenesis of keloid.

The expression levels of ATF3 in the healthy controls, keloid tissue and the normal skin were determined by real-time PCR (a), western blot (b) and immunohistochemistry (c, 400X). *** P < 0.001.

### ATF3 promotes keloid fibroblast proliferation and collagen production

**Figure 2.**
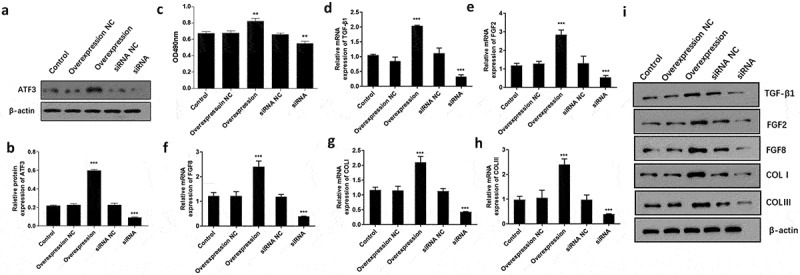
ATF3 promotes cell proliferation and collagen production in keloid fibroblast cells

To examine the functional relevance of ATF3 in keloid fibroblasts, we evaluated the effect of overexpressing or downregulating ATF3 in fibroblast cells. Firstly, ATF3 was successfully upregulated in fibroblast cells upon transfection with the recombinant plasmids encoding ATF3, or downregulated following transfection with siRNA targeting ATF3 (Figure 2(a,b)). As a result, MTT assay showed that ATF3 up-regulation significantly promoted cell viability compared to the control group, whereas transfection with siRNA targeting ATF3 led to an inhibition of cell viability in keloid fibroblast cells (Figure 2(c)). TGF-β1, FGF2 and FGF8 are critical regulator in fibroblast activation [15,17]. Moreover, up-regulation of ATF3 significantly elevated the mRNA and protein levels of TGF-β1, FGF2, and FGF8, while inhibition of ATF3 decreased TGF-β1, FGF2, and FGF8 levels in keloid fibroblast cells (Figure 2(d-f)). Moreover, we found that ATF3 obviously promoted collagen production via elevating COLⅠ and COLⅡⅠ levels in keloid fibroblast cells (Figure 2(g,h)). Moreover, a similar expression pattern of these proteins was observed in fibroblast cells overexpressing or downregulating ATF3 (Figure 2(i)). Collectively, these findings suggest that ATF3 could promote cell growth and collagen production in keloid fibroblast cells.

(a and b) Western blot confirmed that ATF3 was upregulated or downregulated in keloid fibroblast cells following transfection with recombinant vectors encoding ATF3 or siRNA targeting ATF3. (c) The cell viability of fibroblast cells was evaluated by MTT assay in different groups. Real-time PCR was performed to determine the mRNA expression of TGF-β1 (d), FGF2 (e), FGF8 (f), COLⅠ (g) and COLⅠ (h). (i) Western blot was conducted to examined the protein levels of TGF-β1, FGF2, FGF8, COLⅠ and COLⅠ in different groups. ** P < 0.01; *** P < 0.001, compared to control.

### ATF3 suppresses apoptosis in keloid fibroblast cells

**Figure 3.**
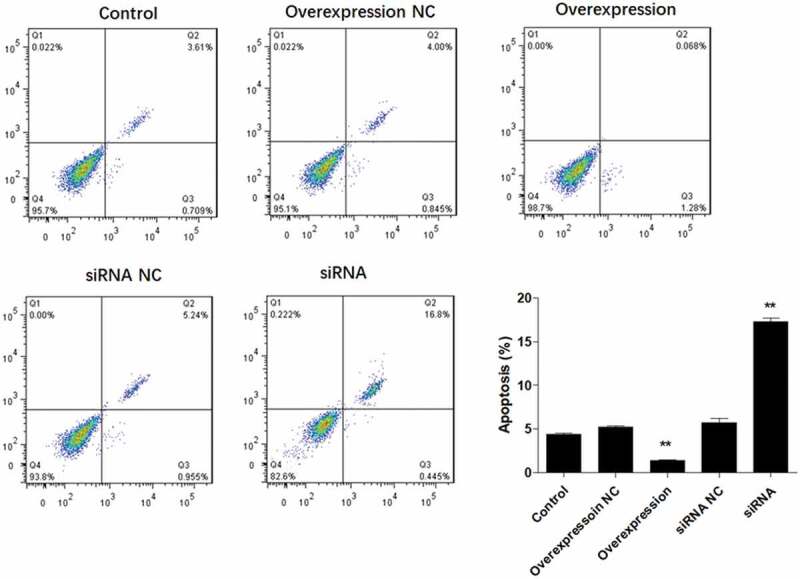
ATF3 suppresses apoptosis in keloid fibroblast cells

**Figure 4.**
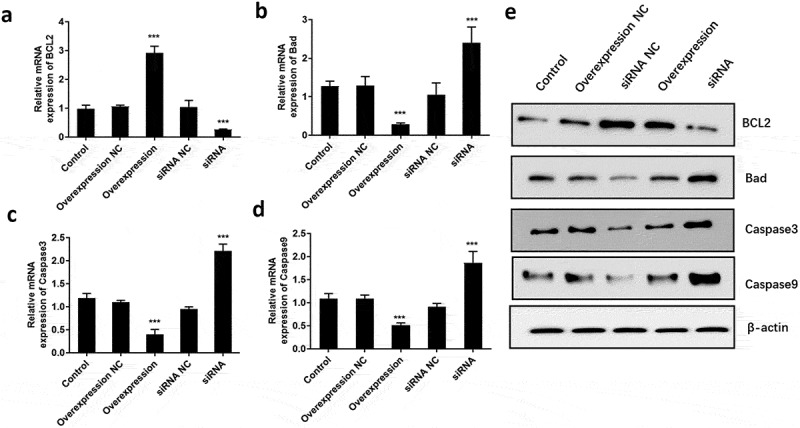
ATF3 inhibited the expression of pro-apoptosis factors in keloid fibroblast cells

It is well known that apoptosis is associated with proliferative ability. Therefore, we further explored the role of ATF3 on fibroblast cell apoptosis. Flow cytometric analysis showed that ectopic expression of ATF3 significantly reduced the numbers of apoptotic cells compared to that of the control group, while inhibition of ATF3 increased the apoptotic rate of keloid fibroblast cells (Figure 3). In addition, we evaluated the mRNA and protein levels of apoptosis-related genes, including BCL2, Bad, Caspase3 and Caspase9 [18], in keloid fibroblast cells. Consistently, real-time PCR analysis showed that ATF3 up-regulation significantly promoted BCL2 mRNA and inhibited mRNA levels of Bad, Caspase3 and Caspase9 (Figure 4(a-d)) in keloid fibroblast cells. By contrast, transfection with siRNA targeting ATF3 led to an inhibition of BCL2 mRNA and upregulation of mRNA levels of Bad, Caspase3 and Caspase9 in keloid fibroblast cells (Figure 4(a-d)). Moreover, a similar expression pattern of these proteins was observed in fibroblast cells overexpressing or downregulating ATF3 (Figure 4(e)). Collectively, these data suggest that ATF3 could suppress the apoptosis rate in the keloid fibroblast cells.

Keloid fibroblast cells were transfected with recombinant vectors encoding ATF3 or siRNA targeting ATF3. Flow cytometry was used to detect the cell apoptosis. In addition, real-time PCR (b) and western blot (c) were conducted to detect the mRNA and protein expression of BCL2, Bad, Caspase3 and Caspase9. *** P < 0.001, compared to control.

Keloid fibroblast cells were transfected with recombinant vectors encoding ATF3 or siRNA targeting ATF3. Real-time PCR was conducted to detect the mRNA expression of BCL2 (a), Bad (b), Caspase3 (c) and Caspase9 (d) in fibroblast cells. Western blot (e) was performed to determine the protein expression of BCL2, Bad, Caspase3 and Caspase9 in fibroblast cells. *** P < 0.001, compared to control.

### ATF3 promotes the invasive potential of keloid fibroblast cells

**Figure 5.**
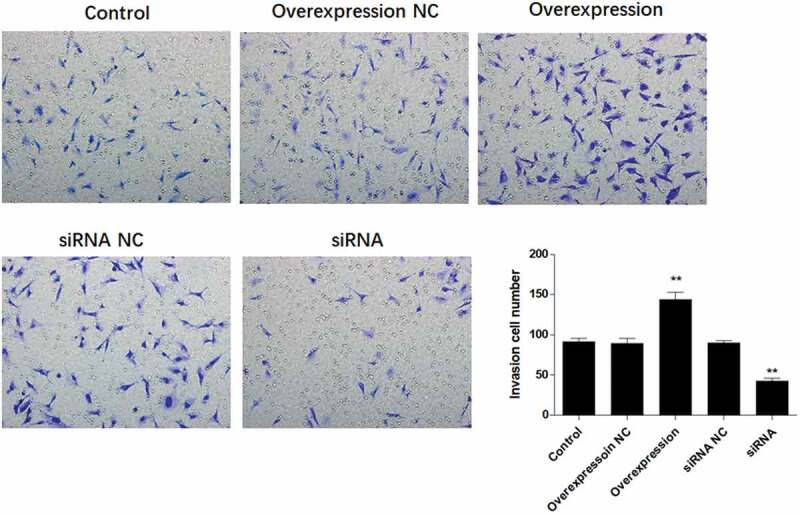
ATF3 promotes the invasive potential of keloid fibroblast cells

**Figure 6.**
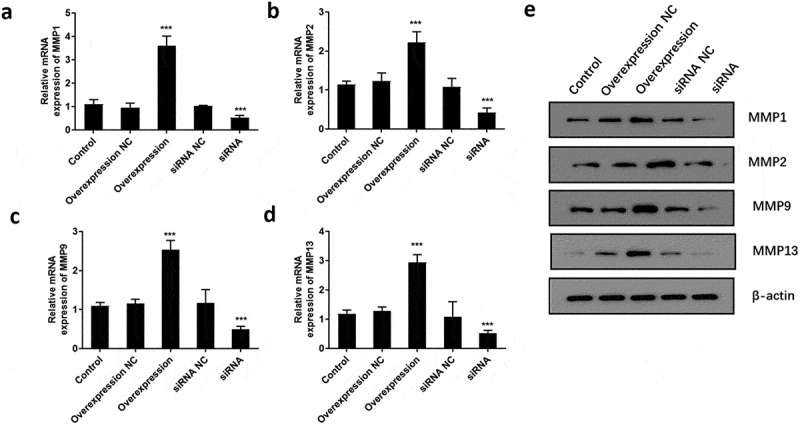
ATF3 promotes the expression of MMPs in keloid fibroblast cells

To determine if ATF3 affected the invasiveness of keloid fibroblast cells, transwell assay was conducted after overexpression or downregulation of ATF3. Subsequently, enforced expression of ATF3 elevated the number of cells with invasive potential; meanwhile, ATF3 siRNA-transfected cells presented with fewer invasive cell number compared to that of the control group (Figure 5). In addition, the mRNA levels of MMP1, MMP2, MMP9, and MMP13 were elevated in ATF3-overexpressing cells and downregulated in fibroblast cells transfected with siRNA targeting ATF3 (Figure 6(a-d)). Consistently, western blot analysis showed that up-regulation of ATF3 significantly promoted levels of MMP1/2/9/13, whereas siRNA ATF3 led to an inhibition of MMP1/2/9/13 in keloid fibroblast cells (Figure 6(e)). Collectively, these data suggest that ATF3 could elevate the expression of MMPs and promote the invasion of keloid fibroblast cells.

Keloid fibroblast cells were transfected with recombinant vectors encoding ATF3 or siRNA targeting ATF3. Transwell invasion assay was used to evaluate the invasive potential of keloid fibroblast cells. Moreover, real-time PCR (b) and western blot (c) were conducted to detect the mRNA and protein expression of MMP1, MMP2, MMP9 and MMP13. *** P < 0.001, compared to control.

Keloid fibroblast cells were transfected with recombinant vectors encoding ATF3 or siRNA targeting ATF3. Real-time PCR was performed to determine the mRNA expression of MMP1 (a), MMP2 (b), MMP9 (c) and MMP13 (d) in fibroblast cells. Western blot (e) was conducted to detect the protein expression MMP1/2/8/13 in fibroblast cells. *** P < 0.001, compared to control.

### ATF3 activates the TGF-β/Smad signaling pathway

**Figure 7.**
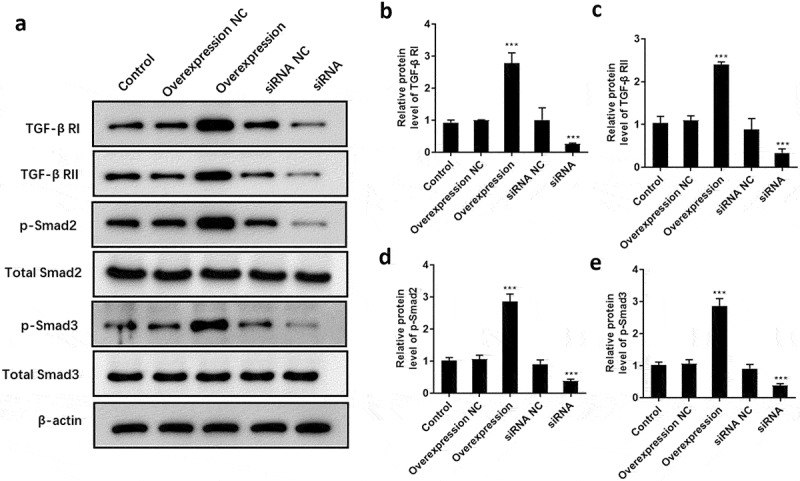
ATF3 promotes the activation of TGF-β/Smad signaling pathway in keloid fibroblast cells

To explore the underlying mechanism by which ATF3 regulates cell proliferation, apoptosis and invasion of keloid ﬁbroblasts, the expression pattern of critical components of TGF-β/Smad signaling was detected. Consequently, data showed that overexpression of ATF3 dramatically promoted the protein levels of TGF-β RI (Figure 7(a,b)) and RII (Figure 7(a,c)), as well as the phosphorylation of Smad2 (Figure 7(a,d)) and Smad3 (Figure 7(a,e)). By contrast, interference of ATF3 decreased the expression of TGF-β RI, TGF-β RII, p-Smad2, and p-Smad3 (Figure 7(a-e)), suggesting that ATF3 exhibited its biological role via regulating TGF-β/Smad pathway in fibroblasts.

Keloid fibroblast cells were transfected with recombinant vectors encoding ATF3 or siRNA targeting ATF3. (a) Western blot was conducted to detect the protein levels of TGF-β RI, TGF-β RII, p-Smad2, p-Smad3, total Smad2, and total Smad3. Quantification of protein signals of TGF-β RI (b) TGF-β RII (c), p-Smad2 (d), and p-Smad3 (e). *** P < 0.001, compared to control.

## Discussion

Pathological keloid will result in a deformed appearance, but the molecular mechanism underlying the keloids has not been fully elucidated. In the present study, we detected the expression pattern of ATF3 in normal skins and keloid tissues, and evaluated its functional roles in keloid fibroblast behaviors. Data showed that ATF3 was dramatically up-regulated in keloid tissues. In addition, downregulation of ATF3 could suppress cell growth and invasion, promoted cell apoptosis, and inhibit collagen synthesis in keloid ﬁbroblasts, suggesting that ATF3 serves as a novel target for the management of keloids.

Accumulating evidence suggests that ATF3 expression is upregulated by a variety of extra- and intra-cellular stimulators [12,13]. Most studies focus on the role of ATF3 in cancers and validate its critical effects on cell growth, apoptosis, cell cycle arrest, metastasis, and acquired drug resistance in a variety of tumor cells [19–22]. ATF3 deficiency has been linked to the spontaneous tumorigenesis in an animal model, which is mediated by genome instability [23]. In breast cancer, ATF3 is involved in regulating the proapoptotic effect of chemotherapeutic drugs [20]. Changes in ATF3 expression are also linked to the activation of inflammatory response. Moreover, ATF3 could enhance macrophage migration and M1/M2 polarization by inducing activation of Wnt/β‑catenin pathway [24]. In cardiac fibroblast cells, ATF3 directly promotes cell proliferation via regulating apoptosis/proliferation-related genes [25]. However, the expression pattern and biological role of ATF3 in keloid fibroblasts remain unknown. In the present study, we found that mRNA and protein expression of ATF3 was significantly elevated in keloid tissues as compared with that of normal healthy controls and parakeloidal skin tissues, suggesting that ATF3 may be involved in the pathogenesis of keloid tissues. Subsequent functional assays showed that ATF3 promoted cell proliferation and collagen production in keloid fibroblast cells. Conversely, transfection with siRNA targeting ATF3 led to decreased cell viability and collagen synthesis via inhibiting TGF-β1 and FGF2/8 production in keloid fibroblasts. The positive roles of TGF-β1 and FGF2/8 in the proliferation and collagen production of fibroblasts have been previously reported [26]. Moreover, ATF3 could reduce the apoptosis rate of keloid fibroblast cells. Molecularly, we found that ATF3 promoted BCL2 level and inhibit the expression of Bad, Caspase3 and Caspase9 in keloid fibroblast cells. It is well known that BCL2 is an anti-apoptotic factor while Bad and Caspase3/9 are contributors to cellular apoptosis [27,28]. Orchestrated changes in cell adhesion molecules and proteolytic enzymes (such as MMP) are associated with the onset of cell invasion in both physiological and pathological situations [29,30]. Furthermore, we found that overexpression of ATF3 enhanced the invasive potential via upregulating the expression of MMP family members, including MMP1, MMP2, MMP9 and MMP13.

It is well documented that TGF-β/Smad pathway plays a critical role in pathological fibrosis [31,32]. Activation of TGF-β/Smad pathway could increase the expression of vascular endothelial growth factor and, in turn, lead to abnormal wound healing [33,34]. In the present study, we observed that ATF3 dramatically induced the activation of TGF-β/Smad signals in fibroblasts, suggesting that ATF3 mediated-fibroblast activation is dependent on TGF-β/Smad pathway.

## Conclusion

The present study provides new insights into the role of ATF3 in human keloid tissues. Our data showed that ATF3 potently promotes growth and invasion, and suppresses apoptosis in keloid fibroblasts via activating TGF-β/Smad pathway, suggesting that ATF3 might serve as a novel therapeutic target for the management of keloid.

## Supplementary Material

Supplemental Material

## Highlights

1. ATF3 enhanced cell proliferation and collagen production in keloid fibroblasts.

2. ATF3 promoted cell invasion and reduced apoptosis in keloid fibroblasts.

3. Mechanically, ATF3 induced the activation of TGF-β/Smad pathway in fibroblasts.

## References

[1] Khalid FA, Mehrose MY, Saleem M, et al. Comparison of efficacy and safety of intralesional triamcinolone and combination of triamcinolone with 5-fluorouracil in the treatment of keloids and hypertrophic scars: randomised control trial. Burns. 2019;45:69–75. 30340861 10.1016/j.burns.2018.08.011

[2] Lee HJ, Jang YJ. Recent understandings of biology, prophylaxis and treatment strategies for hypertrophic scars and keloids. Int J Mol Sci. 2018;19:711. 10.3390/ijms19030711 PMC5877572 29498630

[3] Kafka M, Collins V, Kamolz LP, et al. Evidence of invasive and noninvasive treatment modalities for hypertrophic scars: a systematic review. Wound Repair Regen. 2017;25:139–144. 28056485 10.1111/wrr.12507

[4] Zhou H, Li N, Yuan Y, et al. Activating transcription factor 3 in cardiovascular diseases: a potential therapeutic target. Basic Res Cardiol. 2018;113:37. 30094473 10.1007/s00395-018-0698-6

[5] Rohini M, Haritha Menon A, Selvamurugan N. Role of activating transcription factor 3 and its interacting proteins under physiological and pathological conditions. Int J Biol Macromol. 2018;120:310–317. 30144543 10.1016/j.ijbiomac.2018.08.107

[6] Jadhav K, Zhang Y. Activating transcription factor 3 in immune response and metabolic regulation. Liver Res. 2017;1:96–102. 29242753 10.1016/j.livres.2017.08.001 PMC5724780

[7] Li Y, Liu B, Liang C. Letter by li et al regarding article, “cardiac fibroblast-specific activating transcription factor 3 protects against heart failure by suppressing map2k3-p38 signaling”. Circulation. 2017;136:2092–2093. 29158220 10.1161/CIRCULATIONAHA.117.029716

[8] Li X, Zang S, Cheng H, et al. Overexpression of activating transcription factor 3 exerts suppressive effects in hepg2 cells. Mol Med Rep. 2019;19:869–876. 30535500 10.3892/mmr.2018.9707 PMC6323204

[9] Badr G, Sayed LH, Omar HEM, et al. Camel whey protein protects b and t cells from apoptosis by suppressing activating transcription factor-3 (atf-3)-mediated oxidative stress and enhancing phosphorylation of akt and ikappab-alpha in type i diabetic mice. Cell Physiol Biochem. 2017;41:41–54. 28142150 10.1159/000455935

[10] Wang T, He R, Zhao J, et al. Negative pressure wound therapy inhibits inflammation and upregulates activating transcription factor-3 and downregulates nuclear factor-kappa B in diabetic patients with foot ulcerations. Diabetes Metab Res Rev. 2017;33(4). 10.1002/dmrr.2871 27883358

[11] Qian L, Zhao Y, Guo L, et al. Activating transcription factor 3 (atf3) protects against lipopolysaccharide-induced acute lung injury via inhibiting the expression of tl1a. J Cell Physiol. 2017;232:3727–3734. 28177121 10.1002/jcp.25849

[12] Kim DE, Procopio MG, Ghosh S, et al. Convergent roles of ATF3 and CSL in chromatin control of cancer-associated fibroblast activation. J Exp Med. 2017 Aug 7;214(8):2349–2368. 28684431 10.1084/jem.20170724 PMC5551580

[13] Wu C, Lin H, Zhang X. Inhibitory effects of pirfenidone on fibroblast to myofibroblast transition in rheumatoid arthritis-associated interstitial lung disease via the downregulation of activating transcription factor 3 (ATF3). Int Immunopharmacol. 2019 Sep;74:105700. 31228816 10.1016/j.intimp.2019.105700

[14] Zu T, Wen J, Xu L, et al. Up-regulation of activating transcription factor 3 in human fibroblasts inhibits melanoma cell growth and migration through a paracrine pathway. Front Oncol. 2020 Apr 21;10:624. 32373541 10.3389/fonc.2020.00624 PMC7187895

[15] Meng XM, Nikolic-Paterson DJ, Lan HY. TGF-β: the master regulator of fibrosis. Nat Rev Nephrol. 2016 Jun;12(6):325–338. 27108839 10.1038/nrneph.2016.48

[16] Cui J, Jin S, Jin C, et al. Syndecan-1 regulates extracellular matrix expression in keloid fibroblasts via TGF-β1/Smad and MAPK signaling pathways. Life Sci. 2020 Aug 1;254:117326. 31954164 10.1016/j.lfs.2020.117326

[17] Akatsu Y, Takahashi N, Yoshimatsu Y, et al. Fibroblast growth factor signals regulate transforming growth factor-β-induced endothelial-to-myofibroblast transition of tumor endothelial cells via Elk1. Mol Oncol. 2019 Aug;13(8):1706–1724. 31094056 10.1002/1878-0261.12504 PMC6670013

[18] Lv W, Ren Y, Hou K, et al. Epigenetic modification mechanisms involved in keloid: current status and prospect. Clin Epigenetics. 2020 Nov 26;12(1):183. 33243301 10.1186/s13148-020-00981-8 PMC7690154

[19] Kwon JW, Kwon HK, Shin HJ, et al. Activating transcription factor 3 represses inflammatory responses by binding to the p65 subunit of nf-kappaB. Sci Rep. 2015;5:14470. 26412238 10.1038/srep14470 PMC4585983

[20] Hasim MS, Nessim C, Villeneuve PJ, et al. Activating transcription factor 3 as a novel regulator of chemotherapy response in breast cancer. Transl Oncol. 2018;11:988–998. 29940414 10.1016/j.tranon.2018.06.001 PMC6039300

[21] Vert A, Castro J, Ribó M, et al. Activating transcription factor 3 is crucial for antitumor activity and to strengthen the antiviral properties of onconase. Oncotarget. 2017;8:11692–11707. 28035074 10.18632/oncotarget.14302 PMC5355296

[22] Li X, Zhou X, Li Y, et al. Activating transcription factor 3 promotes malignance of lung cancer cells in vitro. Thorac Cancer. 2017;8:181–191. 28239957 10.1111/1759-7714.12421 PMC5415490

[23] Wang Z, He Y, Deng W, et al. Atf3 deficiency promotes genome instability and spontaneous tumorigenesis in mice. Oncogene. 2018;37:18–27. 28869597 10.1038/onc.2017.310 PMC6179156

[24] Sha H, Zhang D, Zhang Y, et al. Atf3 promotes migration and m1/m2 polarization of macrophages by activating tenascinc via wnt/betacatenin pathway. Mol Med Rep. 2017;16:3641–3647. 28714032 10.3892/mmr.2017.6992

[25] Li YL, Hao WJ, Chen BY, et al. Cardiac fibroblast-specific activating transcription factor 3 promotes myocardial repair after myocardial infarction. Chin Med J (Engl). 2018;131:2302–2309. 30246716 10.4103/0366-6999.241794 PMC6166466

[26] Morris E, Chrobak I, Bujor A, et al. Endoglin promotes tgf-beta/smad1 signaling in scleroderma fibroblasts. J Cell Physiol. 2011;226:3340–3348. 21344387 10.1002/jcp.22690 PMC3381731

[27] Li Y, Liu H, Liang Y, et al. Dkk3 regulates cell proliferation, apoptosis and collagen synthesis in keloid fibroblasts via tgf-beta1/smad signaling pathway. Biomed Pharmacother. 2017;91:174–180. 28458155 10.1016/j.biopha.2017.03.044

[28] Ashkenazi A, Fairbrother WJ, Leverson JD, et al. From basic apoptosis discoveries to advanced selective bcl-2 family inhibitors. Nat Rev Drug Discov. 2017;16:273–284. 28209992 10.1038/nrd.2016.253

[29] Yadav L, Puri N, Rastogi V, et al. Matrix metalloproteinases and cancer - roles in threat and therapy. Asian Pac J Cancer Prev. 2014;15:1085–1091. 24606423 10.7314/apjcp.2014.15.3.1085

[30] Zhang J, Li Y, Bai X, et al. Recent advances in hypertrophic scar. Histol Histopathol. 2018;33:27–39. 28560711 10.14670/HH-11-908

[31] Gordon KJ, Blobe GC. Role of transforming growth factor-beta superfamily signaling pathways in human disease. Biochim Biophys Acta. 2008;1782:197–228. 18313409 10.1016/j.bbadis.2008.01.006

[32] Naim R, Naumann A, Barnes J, et al. Transforming growth factor-beta1-antisense modulates the expression of hepatocyte growth factor/scatter factor in keloid fibroblast cell culture. Aesthetic Plast Surg. 2008;32:346–352. 18087663 10.1007/s00266-007-9078-6

[33] Mallano T, Palumbo-Zerr K, Zerr P, et al. Activating transcription factor 3 regulates canonical TGFβ signalling in systemic sclerosis. Ann Rheum Dis. 2016 Mar;75(3):586–592. 25589515 10.1136/annrheumdis-2014-206214

[34] Lei R, Li J, Liu F, et al. HIF-1α promotes the keloid development through the activation of TGF-β/Smad and TLR4/MyD88/NF-κB pathways. Cell Cycle. 2019 Dec;18(23):3239–3250. 31645185 10.1080/15384101.2019.1670508 PMC6927730

