# Supplementary figures and images for: Activating transcription factor 3 (ATF3) regulates cell growth, apoptosis, invasion and collagen synthesis in keloid fibroblast through transforming growth factor beta (TGF-beta)/SMAD signaling pathway
Source: Bioengineered. 2020 Dec 23;12(1):117–26. doi: 10.1080/21655979.2020.1860491 (PMC8806324; doi:10.1080/21655979.2020.1860491)

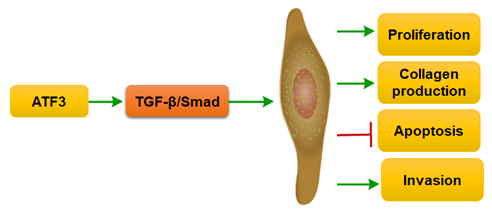

Supplement: Supplemental Material [file KBIE_A_1860491_SM3200.tif]
